# La fracture de Hahn Steinthal traitée par vissage d'Herbert: 3 cas

**DOI:** 10.11604/pamj.2015.20.30.4196

**Published:** 2015-01-13

**Authors:** Bouhelo-Pam Kevin Parfait Bienvenu, El Rhazi Amine, Chmali Khalid, Azarkane Mohamed, El Idrissi Mohamed, Shimi Mohamed, El Ibrahimi Abdelhalim, El Mrini Abdelmajid

**Affiliations:** 1 Service de Chirurgie Ostéo-Articulaire B4, CHU Hassan II, Fès, Maroc

**Keywords:** Capitulum, fracture de Hahn-Steinthal, vissage d′Herbert, ostéosynthèse interne, Capitulum, Hahn-Steinthal fracture, Herbert screw, internal osteosynthesis

## Abstract

Les fractures du capitulum sont rares. Leur prise en charge initiale doit être précoce et efficace en raison des risques engendrés sur le coude: rigidité, instabilité, arthrose. De nombreux traitements ont été proposés. Notre étude décrit le vissage par vis d'Herbert pratiqué chez trois patientes recensées entre 2012 et 2013. Elles ont été inclues selon les critères de traumatisme du coude avec douleur exquise externe avec un trait de fracture radiologique frontal du condyle huméral externe emportant la joue externe de la trochlée. Le diagnostic a été orienté par l'examen clinique et confirmé à la radiographie de face, de profil et des ¾ internes. Les lésions ont été classées selon Bryan et Morrey. Les patients ont été opérés en urgence par abord postéro-latéral de Kocher, réduction à ciel ouvert puis stabilisation par vis de Herbert enfouies. Le recul moyen a été de un an. La récupération fonctionnelle totale moyenne a été de 3,6 mois. L’évaluation fonctionnelle a été jugée excellente selon le score MEPI (Mayo Elbow Performance Index) pour les trois patients. Il n'y a pas eu de démontage de matériel. La consolidation osseuse moyenne a été de 2,6 mois.

## Introduction

Les fractures isolées du capitulum sont rares. Elles représentent 1% de toutes les fractures du coude et 6% des fractures de l'humérus [1]. La prise en charge initiale de ces lésions doit être précoce et efficace en raison des risques engendrés sur le coude à court terme: rigidité, instabilité; et ceux à long terme: arthrose post-traumatique [2]. De nombreux traitements ont été proposés [2]: traitement radical comme l'excision précoce du capitulum et traitement conservateur notamment l'ostéosynthèse par différents implants. Notre étude décrit le vissage par vis d'Herbert afin de contribuer à améliorer la prise en charge de ces lésions.

## Méthodes

Notre étude prospective étendue sur deux ans (2012 et 2013) a permis de retenir trois patients. Ils ont été inclus selon les critères de traumatisme du coude avec douleur exquise externe avec un trait de fracture radiologique frontal du condyle huméral externe emportant la joue externe de la trochlée. Le diagnostic a été orienté par l'examen clinique et confirmé à la radiographie de face, de profil et des ¾ internes. Les lésions ont été classées selon Bryan et Morrey [2]. Les patients ont été opérés en urgence par abord postéro-latéral de Kocher [3], réduction à ciel ouvert puis stabilisation par vis de Herbert enfouies. La mobilité per-opératoire a été appréciée à la recherche d'instabilité. Le recul moyen a été de un an. L’évaluation fonctionnelle a été faite par le score MEPI (Mayo Elbow Performance Index) [4].

## Résultats

### Observation 1

**Figure 1:**
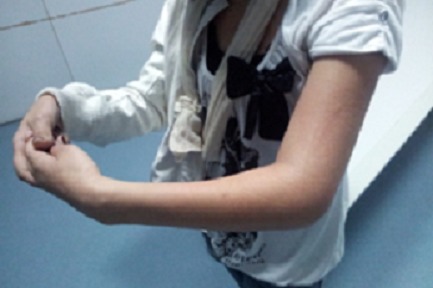
Aspect clinique de coude tuméfié avec attitude traumatique de la patiente 1

**Figure 2:**
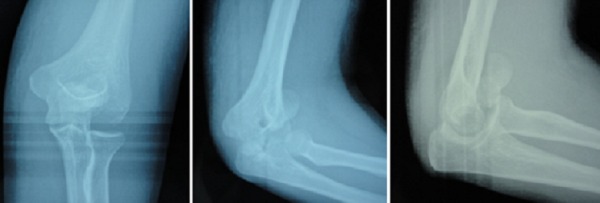
Images radiologiques de face, des ¾ internes, de profil de la patiente 1

**Figure 3:**
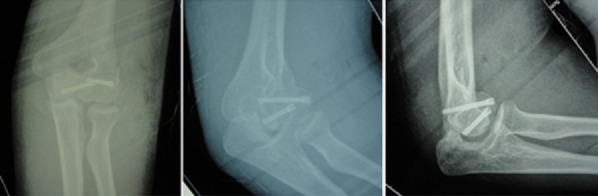
Images radiologiques post-opératoires de face, des ¾ internes, de profil de la patiente 1

Une patiente âgée de 23 ans, droitière, était admise aux urgences à la suite d'une chute de sa hauteur avec réception sur le coude gauche en flexion. A son arrivée, l'examen clinique objectivait une attitude des traumatisés du membre supérieur gauche (Figure 1), une douleur externe du pli du coude gauche avec tuméfaction en regard. Les repères anatomiques du coude étaient conservés et il n'existait pas de déficit sensitif. Les clichés radiographiques (Figure 2) objectivaient une fracture à trait frontal intéressant le capitulum et la joue latérale de la trochlée humérale. La patiente a été opérée en urgence. L'anesthésie était de type bloc axillaire plexique. Après mise en place d'un garrot pneumatique, l'abord du coude a été postéro-latéral selon Kocher [3]. La réduction de la fracture a été obtenue provisoirement par broches de Kirschner contrôlée par amplificateur de brillance. La fixation a été faite par vissage de la trochlée et du capitulum avec 2 vis d'Herbert antéro-postérieures extra-articulaires enfouies dans l'os sous-chondral (Figure 3). Après vérification de la mobilité du coude, une immobilisation complémentaire du coude par attelle postérieure brachio-antébrachiale était mise en place pour une durée de trois semaines. Aucun incident per ou post-opératoire immédiat n'a été noté, la patiente a quitté l'hôpital 24 heures après la chirurgie. La période de suivie a été de un an avec examens clinique et radiologique réguliers. La patiente a été interrogée sur la douleur et les activités quotidiennes. Les mobilités ont été comparées par rapport au coté opposé. Une mobilité réduite a été noté à l'ablation de l'attelle. La rééducation a été débutée à 4 semaines par mobilisations progressives. Les amplitudes articulaires ont été restaurées à 3 mois. Le score MEPI (Mayo Elbow Performance Index) a été de 96. Les signes radiologiques de consolidation ont été partiels à 5 semaines et complets à 2 mois. Il n'y a pas eu de démontage de matériel.

### Observation 2

**Figure 4:**
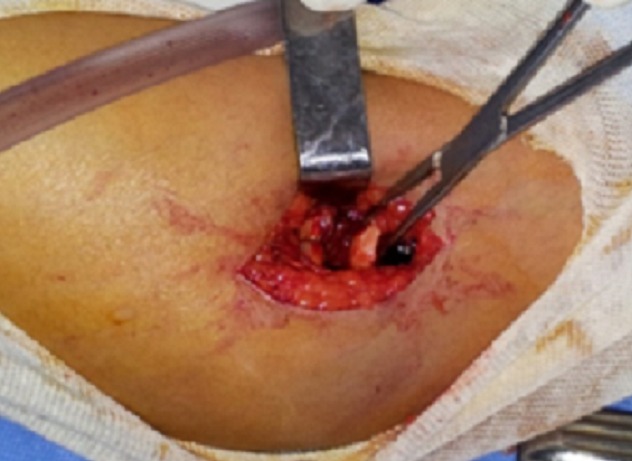
Images per-opératoires de la patiente 2

**Figure 5:**
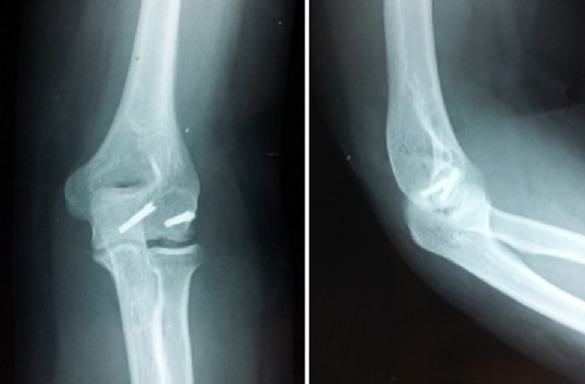
Images radiologiques post-opératoires de face et de profil de la patiente

**Figure 6:**
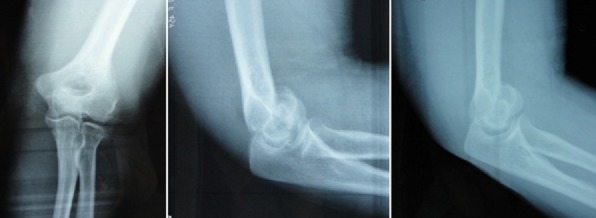
Images per-opératoires de la patiente 3 illustrant le fragment du capitellum à gauche et le fragment de la tête radiale à droite

Une patiente âgée de 21 ans, femme au foyer, droitière, était admise à la suite d'une chute sur le coude gauche, pour douleurs et impotence fonctionnelle. L'examen initial révélait un coude tuméfié, chaud, douloureux, avec douleur exquise antéro-externe. Les repères anatomiques étaient conservés. Le bilan radiologique (Figure 4) objectivait une fracture déplacée du capitellum et de la joue latérale de la trochlée à trait frontal, simple. La patiente a été opérée (Figure 5, Figure 6) en urgence selon les mêmes principes que précédemment. La récupération fonctionnelle a été obtenue au bout de 3 mois également avec un score MEPI à 98. La consolidation osseuse a été complète à 3 mois. Il n'y a pas eu de démontage de matériel.

### Observation 3

**Figure 7:**
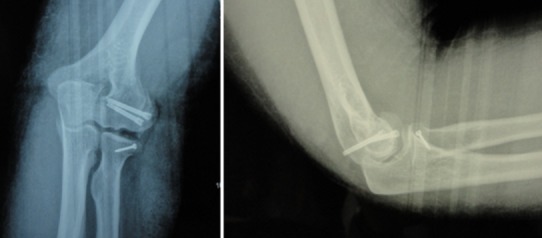
Images radiologiques post-opératoires de face et de profil de la patiente 3

Une patiente âgée de 48 ans, droitière, caissière de profession, était victime d'une chute des escaliers avec réception sur le coude gauche. Elle a été admise aux urgences pour des douleurs du coude gauche avec une impotence fonctionnelle totale. L'examen retrouvait un coude gauche tuméfié avec une ecchymose à la face externe, des douleurs exquises externes. Les repères anatomiques du coude gauche étaient conservés. Les radiographies du coude (Figure 7) objectivaient une fracture du capitellum et de la joue externe de la trochlée ainsi qu'une fracture simple déplacée de tête radiale homolatérale. L'abord postéro-latéral de Kocher au bloc opératoire après bloc anesthésique axillaire plexique a objectivé les deux fractures. Une réduction dans un premier temps de la fracture du capitellum puis vissage par deux vis d'Herbert enfouies ont été effectués. Puis dans un second temps, une réduction de la fracture de la tête radiale puis un vissage par mini-vis à tête. La récupération complète des amplitudes articulaires a été obtenue à 5 mois. Le score MEPI a été de 94. La consolidation osseuse a été complète à 3 mois.

## Discussion

Les rares fractures de Hahn Steinthal rapportées décrivent une prédominance féminine dominée par les patients âgés de plus de 12 ans [5, 6]. Les facteurs favorisants rencontrés sont le cubitus-valgus et le cubitus-recurvatum [7, 8]. Les causes décrites dans la littérature et aussi pour nos patientes sont les chutes sur le coude fléchi. Ce qui résulte de la transmission d'une force axiale à travers le radius qui réalise un cisaillement du capitulum dans le plan coronal [9]. Les trois patientes présentées dans notre étude ont été recensées dans notre service au cours de la période de Janvier 2012 à Décembre 2013, soit deux ans. Le signe d'appel typique est la douleur avec parfois un gonflement latéral du coude. Comme dans notre cas, la radiographie de face peut être normale. Le diagnostic est ainsi posé par les clichés de profil qui montrent un fragment en forme de « croissant de Lune » détaché du condyle huméral. Une TDM avec reconstruction éventuelle peut être réalisée pour l’étude de la taille du fragment et la planification opératoire mais elle n'a pas été indispensable dans notre cas. La classification proposée par Bryan et Morrey [2] a permis de classer nos patients au stade I. La patiente 3 a présenté une forme composite associée à une fracture de la tête radiale classée stade II de Mason [10]. Le traitement des fractures déplacées du capitulum suscite des débats [7–9]. La réduction fermée suivie d'une immobilisation plâtrée a été préconisée. Hahn [11] a été le premier à rapporter le résultat non satisfaisant du traitement orthopédique d'une fracture du capitulum à cisaillement coronal. Durant l'autopsie, il a constaté que le capitulum avait été déplacé et uni à la face antérieure de l'humérus provoquant la restriction de la flexion du coude.

Notre traitement a consisté en une réduction à ciel ouvert plus anatomique et en une stabilisation par 2 vis d'Herbert enfouies, permettant ainsi une meilleure compression. Un << testing >> des mobilités a été fait en per-opératoire évaluant ainsi la congruence. Une grande variété de techniques de stabilisations internes ont été décrits comme des fils de Kirschner [1, 12], broches biodégradables [13], agrafes, pinces à os, et vis de compression [1, 8]. Les broches n'offrent pas une forte compression et stabilisation du site de fracture. La plupart de ces méthodes nécessitent une longue période d'immobilisation post-opératoire ce qui interfère avec la récupération fonctionnelle précoce. Les vis d'Herbert ont l'avantage d’être enfouies donc de ne pas irriter les tissus mous. Il n'y a pas de nécessité de retrait de la vis d'où le programme de rééducation commence plus tôt et la récupération fonctionnelle est plus rapide [1]. Le score MEPI pour nos patientes a été globalement excellent traduisant ainsi une bonne récupération fonctionnelle. L'implant est mis en place en dehors de la surface articulaire ce qui réduit la fréquence d'apparition de l'arthrose. Les inconvénients liés aux vis sans tête sont la pseudarthrose et la chondrolyse. Elles exposent l'implant métallique à la tête radiale adjacente et peuvent conduire à l’érosion ou l'arthrite à l'intérieur de l'articulation. Heureusement, les rapports sur le développement de la pseudarthrose sont rares [14].

## Conclusion

La réduction à ciel ouvert et la fixation interne constituent le traitement de choix pour la fracture de Hahn Steinthal. La stabilisation par vis d'Herbert qui est une méthode moderne donne des résultats satisfaisants car elle permet une forte compression inter-fragmentaire, une mobilisation précoce, et ainsi de la récupération fonctionnelle du coude. L'ablation du matériel d'ostéosynthèse est rarement nécessaire.
